# Isolated Middle-Third Clavicle Fracture Causing Horner's Syndrome: A Case Report and Literature Review

**DOI:** 10.3389/fsurg.2021.640900

**Published:** 2021-05-28

**Authors:** Chia-Yu Lin, Hao-Wei Chang, Yu-Hsuan Chang, I-Hao Lin, Hung-Yu Huang, Cheng-Hsien Chang, Hsien-Te Chen, Yi-Wen Chen, Tsung-Li Lin, Chin-Jung Hsu

**Affiliations:** ^1^Department of Orthopedics, China Medical University Hospital, China Medical University, Taichung, Taiwan; ^2^Department of Medical Image, National Taiwan University Hospital, Taipei, Taiwan; ^3^Department of Neurology, China Medical University Hospital, China Medical University, Taichung, Taiwan; ^4^Department of Ophthalmology, China Medical University Hospital, China Medical University, Taichung, Taiwan; ^5^Department of Optometry, College of Medical and Health Science, Asia University, Taichung, Taiwan; ^6^Spine Center, China Medical University Hospital, China Medical University, Taichung, Taiwan; ^7^Department of Sport Medicine, College of Health Care, China Medical University, Taichung, Taiwan; ^8^Graduate Institute of Biomedical Sciences, China Medical University, Taichung, Taiwan; ^9^3D Printing Medical Research Institute, Asia University, Taichung, Taiwan; ^10^School of Chinese Medicine, China Medical University, Taichung, Taiwan

**Keywords:** clavicle fracture, Horner's syndrome, trauma, emergency, oculosympathetic pathway

## Abstract

The pathophysiology of Horner's syndrome arises due to compression or destruction of the oculosympathetic nerve pathway. Traumatic Horner's syndrome may indicate lethal neurovascular injury, such as brain stem lesion, cervical spine injury, or carotid artery dissection. The middle-third is the most common type of clavicle fracture. However, the association of the isolated middle-third clavicle fracture and Horner's syndrome is rare. We report the case of a 47 year-old woman who presented to our emergency department with acute trauma. Severe tenderness and limited mobility were observed in her left shoulder. On radiographic examination, a middle-third clavicle fracture was diagnosed. Ptosis and myosis were also noticed on further examination, and she was subsequently diagnosed with Horner's syndrome. A survey of the brain, cervical spine, carotid artery, and lung revealed no pathological findings. Surgery for the clavicle fracture was performed 2 days after the accident. The patient recovered from Horner's syndrome gradually over the 2 months following the surgery, and the syndrome completely resolved by the third month. To the best of our knowledge, this is the first report of traumatic Horner's syndrome caused by an isolated middle-third clavicle fracture. The improved outcome may be attributed to the surgical intervention for middle-third clavicle fracture, which may help release ganglion or neuronal compression.

## Introduction

Horner's syndrome is characterized by the classic triad of ipsilateral miosis, ptosis, and anhidrosis (1). The pathophysiology of Horner's syndrome arises due to compression or destruction of the oculosympathetic pathway, which includes three neurons: central, pre-ganglion, and post-ganglion, according to the location of damage (2). The first-order, central neuron, starts on the posterior hypothalamus and ends in the intermediolateral horn of the spinal cord, from C8-T2. The second-order, pre-ganglion neuron, is located at the thoracic outlet, lung apex, and the subclavian artery, connects the stellate and middle cervical ganglia and ends in the superior cervical ganglion. The third-order, post-ganglion neuron, exits the superior cervical ganglion and is located near the internal carotid artery. Therefore, Horner's syndrome can result from a variety of etiologies such as brain stem lesions, cervical spine dislocation, carotid artery dissection, lung tumors, thoracic and cervical surgical procedures, clavicle tumors, or trauma (3–10).

The clavicle is a superficial flat bone that is prone to injury during trauma, such as a fall onto an outstretched upper extremity. It accounts for up to 2.6–4% of all fractures and 44–66% of all fractures of the shoulder (11, 12). However, damage to the neurovascular structure, which is usually associated with penetrating traumas, rarely occurs in closed fractures of the clavicle.

In this report, we present the diagnosis and treatment of a rare case of Horner's syndrome caused by middle-third clavicle fracture in a motor vehicle collision, which to the best of our knowledge, is the first report of traumatic Horner's syndrome caused by an isolated middle-third clavicle fracture.

## Case Report

**Figure 1:**
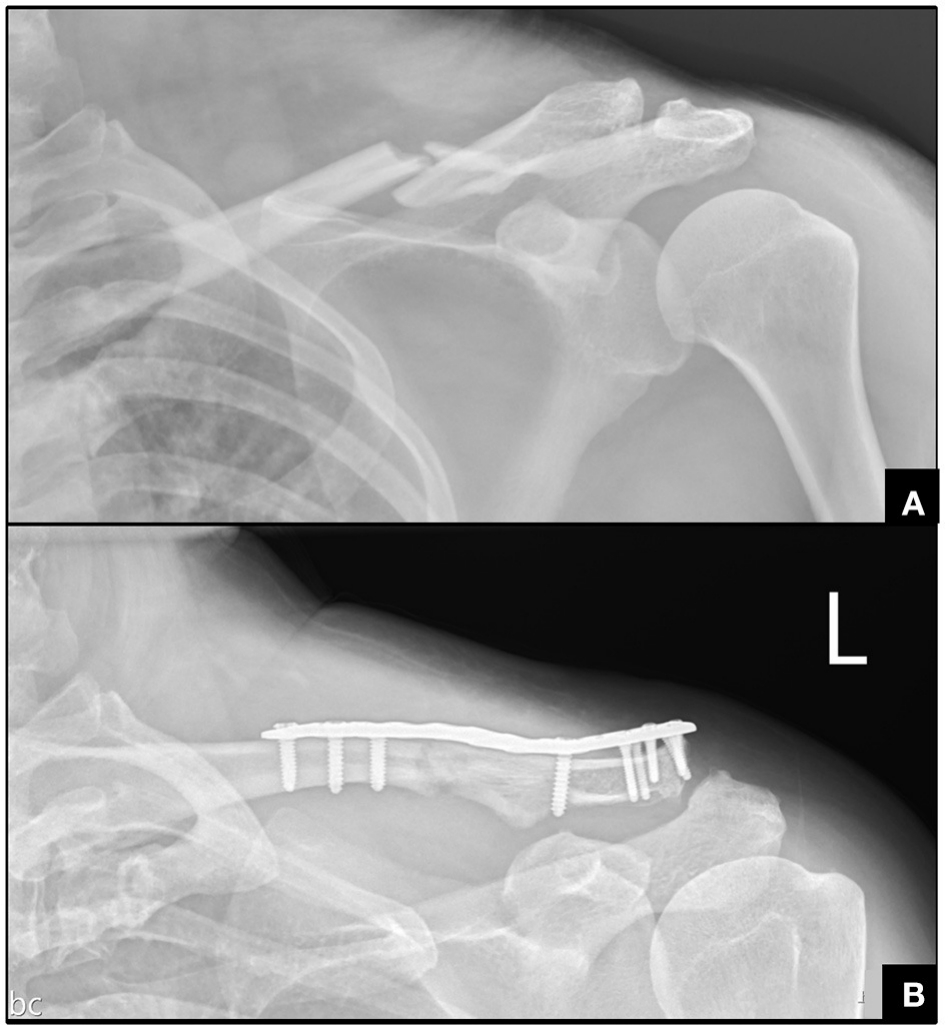
Radiography for the clavicle fracture. **(A)** Left middle-third clavicle fracture. **(B)** Postoperative radiography.

**Figure 2:**
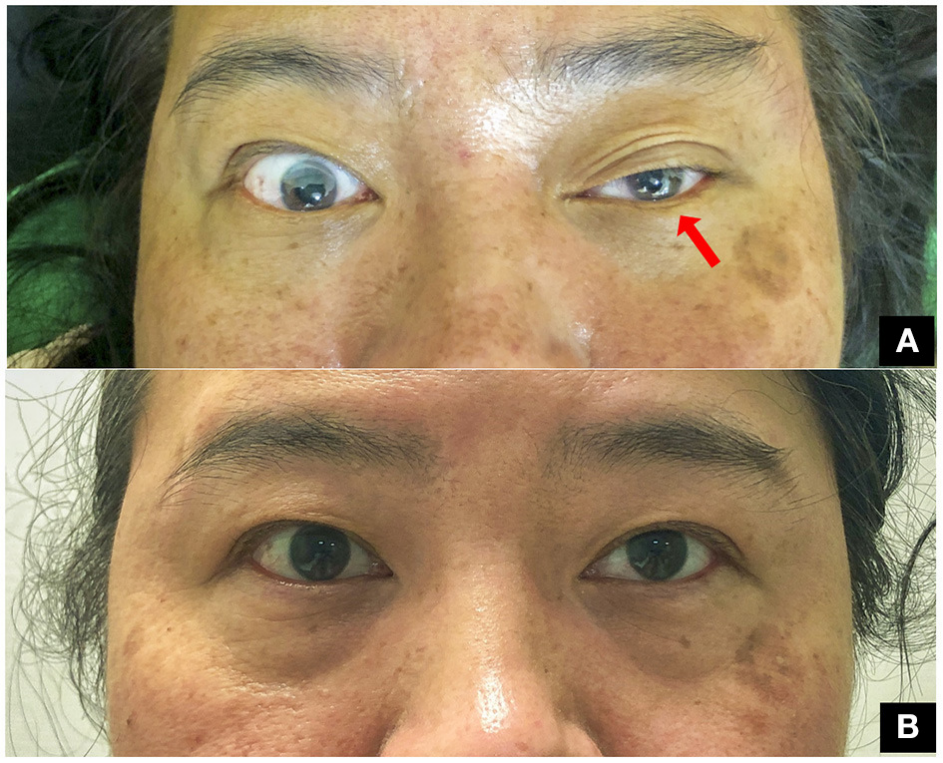
Gross appearance of the patient. **(A)** Ptosis and myosis on the left side observed in the emergency department (room light). **(B)** Symmetrical eye opening and pupil size at outpatient clinic on the third month (room light).

**Figure 3:**
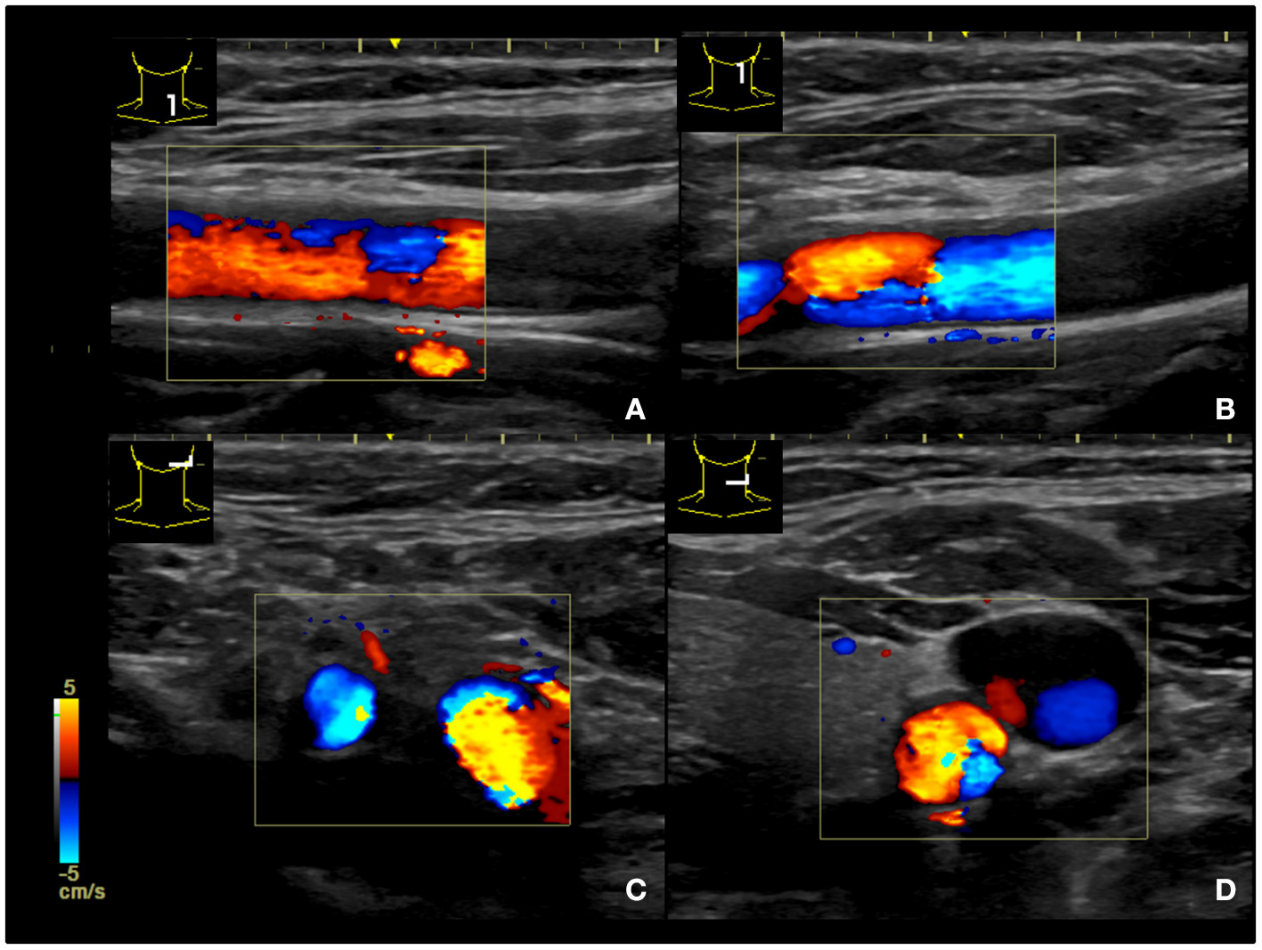
Ultrasound survey of the carotid artery revealing intact structures. **(A,B)** longitudinal plane of the carotid artery. **(C,D)** transverse plane of the carotid artery.

A 47-year-old woman presented to the emergency department after a motor vehicle collision. On admission, she was conscious and hemodynamically stable, with pain in the left chest and shoulder region. Physical examination revealed symmetrical breathing and several abrasion wounds around the left shoulder. The distal neurovascular status of the left upper limb was fair. Radiography revealed a fracture of left middle-third clavicle (Figure 1A). Neither pneumothorax nor hemothorax was noted. However, during a second examination, we found that her left eyelid was asymmetrically dropped. Complete physical and neurological examinations were immediately performed again. Ptosis and miosis on her left eye were discovered (Figure 2A). There was no pupillary response to direct or consensual light, and cardinal fields of gaze and facial sensations were normal. Further, pathologic reflexes were not elicited, and results were normal upon otoscopic examination. The patient's history was negative for medical, neurological, or ophthalmological disorders. As Horner's syndrome was suspected, we performed chest computed tomography (CT), brain magnetic resonance angiography (MRA) and cervical spine magnetic resonance imaging (MRI), which revealed no evidence of tumors, vascular or spinal cord injury. Moreover, ultrasonography of the neck revealed an intact carotid artery (Figure 3). The diagnosis of Horner's syndrome was confirmed after pharmacological testing excluded for the use of cocaine. Following hydroxyamphetamine testing, the lesion was localized to the preganglionic nerve, indicating Horner's syndrome.

Two days after the accident, she received surgery for open reduction and internal fixation of the clavicle fracture (Figure 1B). She denied family history of bleeding diathesis and was not taking any antithrombotic medication. The results of the complete pre-operative survey including a coagulation study were unremarkable. A skin incision was made just above the clavicle bone, and gentle dissection was performed to reach the bone. The soft tissue was swollen, with some hematomas around the fracture site. The fracture site was reduced and then fixed with a plate and screws. Adequate hemostasis was completed before wound closure. No drainage was set. The surgery was performed with no complications. Oral vitamin B12 was administered after surgery. Regular follow-ups in the outpatient department revealed that she recovered a full range of motion and was free of left shoulder pain at the first month post-surgery. During the outpatient follow-up 2 months after the surgery, the pupil sizes had become symmetric and ptosis had improved. The symptoms of Horner's syndrome completely resolved on the third month after clavicle surgery (Figure 2B).

## Discussion and Conclusion

**Figure 4:**
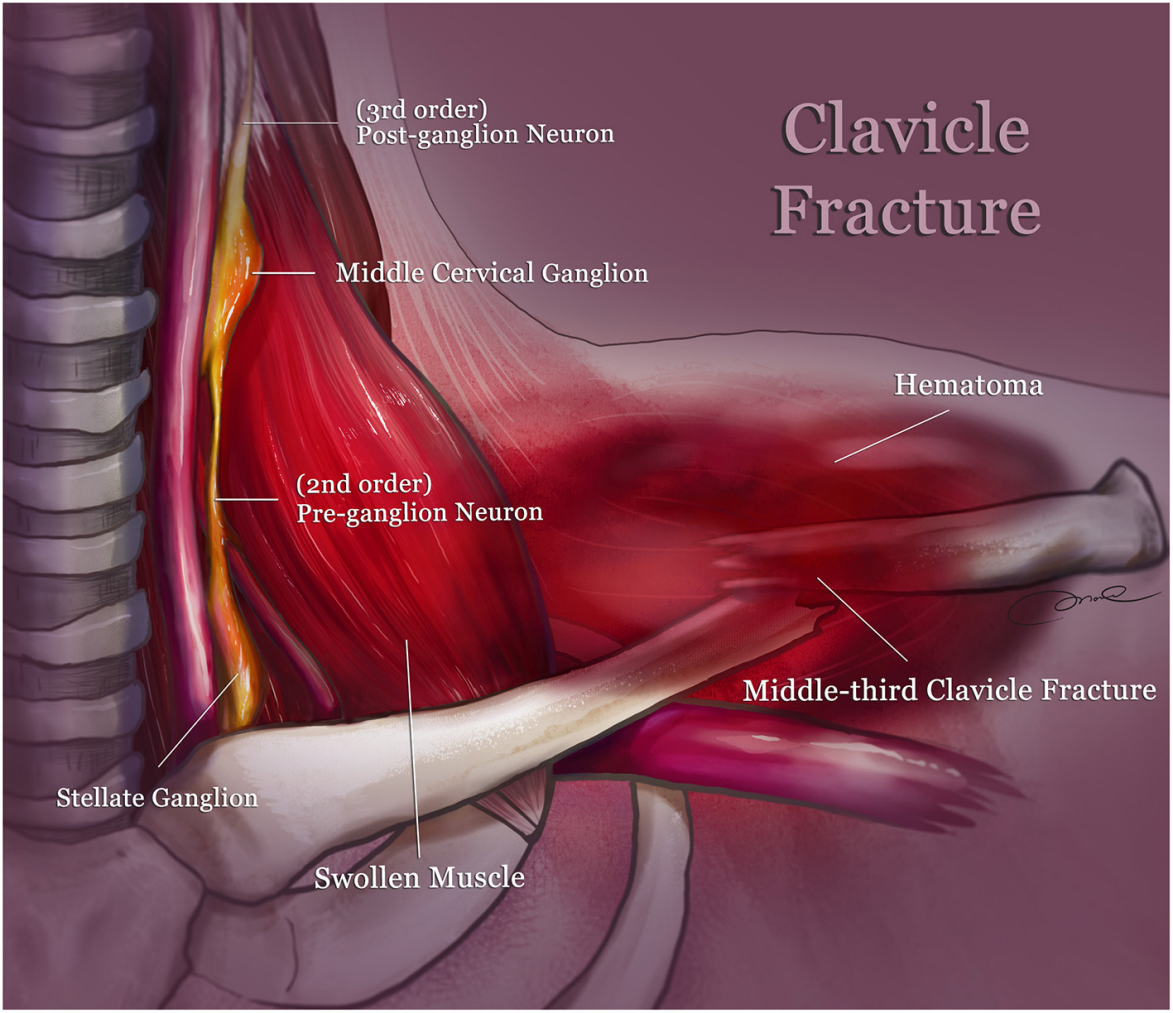
Schematic depicting the injury mechanism: the oculosympathetic pathway may be compressed by the swollen muscle (scalene, sternothyroid muscle) causing a hematoma along the middle-third clavicle fracture. The three neurons involved in this pathway are the first order central neuron (inside the spinal cord, not shown here), second order pre-ganglion neuron (around the thoracic outlet), and third order post-ganglion neuron (near the internal carotid artery).

Horner's syndrome is characterized by several characteristic features, including ipsilateral miosis, ptosis, and anhidrosis (1, 2). The diagnosis can be confirmed by pharmacological testing with the use of cocaine, and the lesion can be localized to the post or non-post neuron using hydroxyamphetamine (2). Traumatic Horner's syndrome can be due to an injury around the oculosympathetic pathway, including the brain stem, cervical spine, or carotid artery (3–5). In this case, complete physical and neurological examinations were performed, and there were no imaging findings on a brain MRA, cervical spine MRI, ultrasonography of the neck, and chest CT. It was therefore concluded that this patient suffered from Horner's syndrome due to an isolated middle-third clavicle fracture, which is a rare etiology. The fracture site was beside the thoracic outlet and relatively close to the lung apex, where the bony fragment, swollen soft tissue, or a hematoma might cause an interruption or compression of the second-order neuron, leading to preganglionic Horner's syndrome (Figure 4). All cases of traumatic Horner's syndrome, or iatrogenic Horner's syndrome, mentioned in the literature are summarized in Table 1 (6–8, 13–24). Karen et al. reported osteochondroma of the clavicle, which, although rare, should be considered in the differential diagnosis of causes of Horner's syndrome (10). A patient with Horner's syndrome caused by compression from the tip of a chest tube recovered in 5 days, up to 2 months after chest tube repositioning (8). In patients who developed Horner's syndrome following a superficial cervical plexus block, recovery was complete after 1.5 h (7). However, most patients who suffered from traumatic Horner's syndrome associated with cervical trauma, rib, or clavicle fracture (6, 13, 15–19, 21, 22), displayed more variability in time to recovery (from 6 weeks to 2 years), and some did not, or only partially, improved. The possible reasons for this are the direct effect of the swollen soft tissue, fractured bone, or hematoma on the pre-ganglion neuronal pathway. The sympathetic neuron may be decompressed after swelling of the soft tissue subsides or the hematoma is absorbed, or the fracture is reduced.

**Table 1:** Summary of the literature review of trauma-related Horner's syndrome.

**Study**	**Diagnosis**	**Cause of Horner's syndrome**	**Management**	**Resolution/follow-up**
Goost et al. (13)	1st rib fracture	NM	Conservative treatment	Complete/6 months
Demetrious (14)	1st rib fracture	NM	Conservative treatment	Complete/1 year
Lin et al. (15)	1st rib fracture	Hematoma	Conservative treatment	Complete/2 years
Ofri et al. (16)	1st rib fracture	NM	Conservative treatment	Complete/6 weeks
Quero et al. (17)	Ipsilateral 1st and 6th rib fractures	Hematoma	Conservative treatment	Incomplete recovery/6 months
Ahmadi et al. (6)	Ipsilateral 1st rib and scapula fractures	NM	Conservative treatment	Complete/8 weeks
Guerra et al. (18)	Ipsilateral 3rd rib and clavicle fractures	NM	Conjunctivo-Müllerectomy	Unimproved/1 year
Moraga et al. (19)	Ipsilateral 1st to 6th rib, scapular, and clavicle fractures	Hematoma	Conservative treatment	Unimproved/6 months
Guillén-Paredes et al. (20)	Cervical penetrating trauma	Cervical penetrating trauma	Conservative treatment	Unimproved/1 year
Asensio-Sánchez et al. (21)	Cervical trauma	Neck hematoma	NM	NM
Bell et al. (22)	Cervical trauma	Carotid artery dissection	Anti-coagulation therapy for brain infarction	Complete/NM
Yang et al. (23)	Internal mammary artery pseudoaneurysm	Pseudoaneurysm	Surgery for the pseudoaneurysm	Residual miosis/6 months
	Internal carotid artery pseudoaneurysm	Pseudoaneurysm	Surgery for the pseudoaneurysm	Unimproved/NM
Kaya et al. (8)	Whole body trauma including 1st rib fracture	NM	NM	Unimproved/9 months
	Whole body trauma including clavicle fracture	NM	NM	Unimproved/6 months
	Whole body trauma including clavicle fracture	NM	NM	Unimproved/9 months
	Whole body trauma including clavicle fracture	NM	NM	Unimproved/6 months
	Cervical trauma, 1st rib and clavicle fractures	NM	NM	Unimproved/1 year
	Pneumothorax post chest tube insertion	Chest tube tip pressure	Chest tube repositioning	Complete recovery/5 days
	Pneumothorax post chest tube insertion	Chest tube tip pressure	Chest tube repositioning	Complete recovery/3 weeks
	Traumatic hemopneumothorax post chest tube insertion	Chest tube tip pressure	Chest tube repositioning	Complete recovery/2 weeks
	Traumatic bronchial rupture post chest tube insertion	Chest tube tip pressure	Chest tube repositioning	Complete recovery/2 months
	Bronchiectasis post chest tube insertion	Chest tube tip pressure	Chest tube repositioning	Incomplete recovery/2 months
Watura et al. (10)	Osteochondroma of the clavicle	Soft tissue compression	Pending surgical excision	Unimproved/3 months
Lin et al. (current study)	Isolated middle-third clavicle fracture	Hematoma	Open reduction and internal fixation	Complete recovery/3 months

*NM, not mentioned*.

By comparing the resolution of traumatic Horner's syndrome caused by chest tube compression to rib fracture, we hypothesize that recovery starts when the neuronal compression is released, and the time period to recovery is associated with the duration of compression. Generally, if the nerve is compressed for a longer period, the time to recovery is longer. None of the patients mentioned in the literature who had Horner's syndrome with a first rib fracture received an operation (6, 14–17, 19), and neither did the patient with a clavicle and third rib fracture (18). Most patients took an extended period to recover, some with no improvement, and one even needed ophthalmological surgery (18). As opposed to rib fracture, which occurs in the stable thoracic cage, a middle-third clavicle fracture usually involves more displacement and is more unstable. Therefore, we suggest earlier fracture reduction and removal of hematoma in cases where traumatic Horner's syndrome is caused by clavicle fracture, as in this reported case.

To the best of our knowledge, this is the first report of a patient who recovered from Horner's syndrome associated with isolated middle-third clavicle fracture. Clinicians should arrange a complete Horner's syndrome examination using ultrasound, MRA, MRI, and CT to rule out other lesions along the oculosympathetic pathway. Although middle-third clavicle fracture can be treated conservatively, surgical management has potential benefit for cases with neurological deficit, such as this case. As soon as the general condition of the patient was stable, internal fixation was performed. Surgery may therefore improve patient outcomes as removing the hematoma or reducing the fracture site will alleviate compression of the impacted ganglion or neuron.

## Data Availability Statement

The raw data supporting the conclusions of this article will be made available by the authors, without undue reservation.

## Ethics Statement

Written informed consent was obtained from the individual(s) for the publication of any potentially identifiable images or data included in this article.

## Author Contributions

C-YL, H-WC, T-LL, Y-HC, and C-JH contributed to the conception and design of the study. C-YL, H-WC, and T-LL contributed to drafting the article. Y-HC, I-HL, T-LL, C-JH, H-TC, H-YH, Y-WC, and C-HC contributed to revising the article critically. All the authors have read and approved the manuscript.

## Conflict of Interest

The authors declare that the research was conducted in the absence of any commercial or financial relationships that could be construed as a potential conflict of interest.
